# How early-career researchers are shaping eLife

**DOI:** 10.7554/eLife.36263

**Published:** 2018-03-27

**Authors:** Mark Patterson, Randy Schekman

**Keywords:** Scientific publishing, Early-career researchers, grad school, postdoc, careers in science, peer review

## Abstract

Journals can benefit from listening to graduate students, postdocs and newly-independent group leaders.

Before eLife published its first article, there was already strong agreement within the initiative that the journal had to appeal to early-career researchers, as well as established scientists. Towards this end we introduced a number of measures that, we hoped, would encourage graduate students, postdocs and new group leaders to submit their work to the journal (Schekman et al., 2013). Now, more than five years on, our relationship with early-career researchers has evolved considerably as they have become more involved in the governance of eLife and discussions about the future of the journal and the eLife initiative more broadly.

The first concrete step that eLife took in this direction was to recruit an Early-Career Advisory Group (ECAG) in 2014. Inspired by perspectives from researchers whose opinions resonated strongly with eLife’s mission, a team of 14 graduate students, postdocs and newly-independent group leaders was assembled. This group has provided insight, ideas and constructive criticism of eLife policies and practices and, as discussed below, has helped to refine and launch several initiatives.

One of the most important outcomes from the ECAG discussions has been the recruitment of a pool of early-career reviewers. It is common practice for busy group leaders to ask their more senior PhD students and postdoctoral fellows to help with peer review, but in too many cases these contributions go unacknowledged. Nevertheless, such experience can help an early-career researcher gain the confidence and judgment to participate in peer review in their own right. Building on this practice and after discussions with the ECAG, we asked our Board of Reviewing Editors to nominate early-career colleagues who they felt were ready to act as independent peer reviewers. We have now amassed a group of more than 250 early-career reviewers; around 100 have peer-reviewed eLife submissions, and have also benefited from the open consultation that takes place between reviewers in the eLife process.

The involvement of the early-career community in eLife’s publishing work is only set to increase as we expand the early-career reviewer pool and find better ways to bring these individuals to the attention of editors when they are looking for referees. In addition, several early-career reviewers have recently become Reviewing Editors for the journal. Another benefit of the early-career reviewer pool is that it is more balanced with respect to gender and geography than the existing editorial community. As well as helping to identify highly competent editors and reviewers, we therefore hope that the reviewer pool will lead to greater diversity in all aspects of our publishing.

We have also appointed an early-career researcher to the eLife Board of Directors. One of the important functions of the Board of a non-profit organization like eLife is to provide oversight of our strategic direction, and to ensure that we stay true to our mission. Early-career researchers are a critical constituency in eLife’s mission, and it therefore makes complete sense to have that constituency represented at the highest levels of governance in our organization.

Another recent development is the eLife Ambassador program, which aims to support the development and reach of initiatives that range from encouraging the use of preprints to increasing the reproducibility of science. Although not limited to early-career researchers, the idea for this program emerged after the election for members of the ECAG in 2017. The response was so enthusiastic that we wanted to provide a way for researchers who support eLife’s goals to work together on areas of common interest. Various working groups have now been set up with support from eLife staff and we hope that these groups will help to forge new connections amongst researchers, as well as ideas for new initiatives aimed at improving the operation of scientific publishing.

We have now amassed a group of more than 250 early-career reviewers

As eLife has grown and become established as a major publication in the life and biomedical sciences, some of the initial approaches that we introduced to support early-career researchers have been adjusted. For example, senior editors would often write letters of support for early-career eLife authors to help with job and funding applications, but such supporting letters are now requested only infrequently. Another policy was to peer review more articles from newly-independent investigators so that these authors could benefit from more detailed feedback. Although a higher proportion of early-career articles were invited for peer review, a higher proportion were also then rejected after peer review. The editors felt that they were not serving the interests of beginning investigators well in this way, and that it was better to assess all initial submissions by the same standards.

Other initiatives have been built upon or launched. The ECAG has helped to refine our travel grant program and has introduced a series of webinars on topics of interest to the early-career community. All of these webinars have been recorded and are building into a valuable resource. Early-career researchers and issues of concern to them are also regularly featured on the Community page, and in interviews, podcasts and articles in the Magazine section of eLife.

eLife’s mission is about the future of science. Whether we are talking about innovations in technology to support greater reproducibility, editorial policies that set the standards for eLife research articles, or the long-term financial sustainability of eLife, early-career perspectives are part of the conversation. Our work at eLife has been enriched by these perspectives, and no doubt so would many other initiatives aimed at improving the culture and communication of research.
